# Effect of DNA density immobilized on gold nanoparticles on nucleic acid detection†

**DOI:** 10.1039/d3ra06528f

**Published:** 2023-10-19

**Authors:** Gen Hirao, Nanami Fukuzumi, Atsushi Ogawa, Tsuyoshi Asahi, Maeda Mizuo, Tamotsu Zako

**Affiliations:** a Department of Chemistry and Biology, Graduate School of Science and Engineering, Ehime University 2-5 Bunkyo Matsuyama Ehime 790-8577 Japan zako.tamotsu.us@ehime-u.ac.jp; b Proteo-Science Center, Ehime University 2-5 Bunkyo Matsuyama Ehime 790-8577 Japan; c RIKEN Cluster for Pioneering Research 2-1 Hirosawa Wako Saitama 351-0198 Japan

## Abstract

Gold nanoparticles (AuNPs) have been utilized as colorimetric biosensors, where target molecule-induced AuNP aggregation can be recognized by a colour change from red to blue. Particularly, single-stranded DNA (ssDNA)-immobilized AuNPs (ssDNA-AuNPs) have been applied to genetic diagnosis due to their rapid and sequence-specific aggregation properties. However, the effect of the density of immobilized ssDNA have not been investigated yet. In this study, we developed a method to control the amount of immobilized ssDNA by use of ethylene glycol, which is expected to control the ice crystal spacing in a freezing-thawing ssDNA-AuNP synthesis method. We also investigated the effect of the DNA density on the sensitivity of the target ssDNA detection, and found that the detection sensitivity was improved at lower DNA densities. To discuss the reason for the improved detection sensitivity, we modified the ssDNA-AuNPs with alkane thiol for better dispersion stability against salt. The results suggest that the DNA density, rather than the dispersion stability, has a significant impact on detection sensitivity.

## Abstract 2

The detection sensitivity of ssDNA was better when gold nanoparticles modified with DNA of lower density were used.

## Graphical abstract

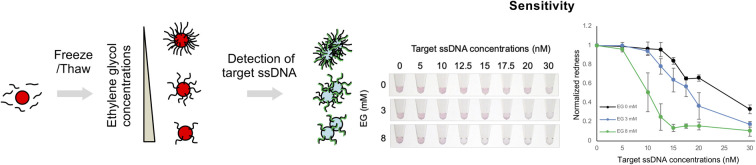

## Introduction

Gold nanoparticles (AuNPs) are nanometre-sized particles with different optical, chemical, and electronic properties depending on their size and shape.^1–4^ In particular, the AuNP solution is red in colour when the NPs are dispersed and purple when they are aggregated due to a change in surface plasmon resonance. If this solution colour change occurs only in the presence of a target molecule, the target molecule can be detected by naked-eyes. Thus AuNP-based colorimetric sensors have been developed to detect various molecules such as biomolecules (*e.g.* DNA and protein), metal ions and organic compounds.^2,5–9^

It was previously shown that single-stranded DNA (ssDNA) could be detected by utilizing the salt-induced non-crosslinking aggregation of the complementary ssDNA-immobilized AuNPs (ssDNA-AuNPs); upon duplex formation on the AuNP surface, double stranded (ds)DNA-AuNPs rapidly aggregate at high salt concentrations, while ssDNA-AuNPs are dispersed due to electrostatic and steric repulsion between particles.^10–12^ We have also reported highly sensitive ssDNA detection using dark-field microscopic observation of the aggregated AuNPs at the single-cluster level.^13,14^ In addition, investigations on the important factors affecting ssDNA-AuNP aggregation such as temperature, probe length and particle size have been reported.^15–17^ However, effect of density of immobilized ssDNA on the AuNP surface on the detection sensitivity of the target ssDNA is still unclear. In this study, we developed a facile method to control the amount of ssDNA immobilized on the AuNP surface, and investigated the effect of the surface density of the immobilized ssDNA on the target ssDNA detection sensitivity.

In this study, we employed a freezing method for the immobilization of the thiolated ssDNA on AuNP surface *via* thiol-Au bonds. Upon freezing, small ice crystals mainly composed of pure water are formed, and non-water species such as AuNPs, DNA and salt are concentrated in the gaps between the ice crystals, leading to a fast immobilization of thiolated ssDNA on the AuNP surface.^18,19^ It is noted that no effect was observed on the size of AuNPs by the freezing–thawing process since the size of ssDNA-AuNPs made by the freezing method and that by the salt-aging method were the same.^18^ It has previously been shown that ethylene glycol (EG) could prevent aggregation of silver nanoparticles by freezing-thawing.^20,21^ EG lowers the vapour pressure of water and the freezing point of the solution, thereby inhibits the formation of ice crystals. Thus we hypothesized that EG could be used to control the amount of ssDNA immobilized on AuNPs. In this study, we demonstrated for the first time that the amount of ssDNA immobilized on AuNPs could be easily controlled using EG in the ssDNA-AuNP synthesis by the freezing method, and investigated the effect of DNA density on the detection of the target ssDNA.

Herein, we found that the detection sensitivity was higher when ssDNA-AuNPs with lower amount of the immobilized ssDNA were used. However, it was not clear that the higher sensitivity was due to lower stability against salt. In order to discuss this result, mercapto-1-hexanol (MCH) was introduced on the surface of ssDNA-AuNPs.^22,23^ Previous study has shown that the alkanethiol modification enhanced the dispersion stability of AuNPs.^24–26^ We successfully made MCH-modified ssDNA-AuNPs with lower amount of the immobilized ssDNA that showed the same salt stability as MCH-free ssDNA-AuNPs with higher amount of the immobilized ssDNA. Since the detection sensitivity was better for the former ssDNA-AuNPs, our results suggest that the amount of immobilized ssDNA is important for the efficient detection. Our study would give a basis for the further improvement of AuNP-based molecular sensing.

## Experimental

### Materials

AuNPs (40 nm) were obtained from BBI solutions (Cardiff, UK). Thiolated ssDNA (probe ssDNA, 5′-SH-TACGCCACCAGCTCC-3′) was purchased from Integrated DNA Technologies (Coralville, IA, USA). Complementary ssDNA (target ssDNA, 5′-GGAGCTGGTGGCGTA-3′) and non-thiolated ssDNA (5′-TACGCCACCAGCTCC-3′) were purchased from Eurofins Genomics (Tokyo, Japan). Dithiothreitol (DTT), and EG were purchased from Wako (Osaka, Japan). MCH was purchased from Sigma-Aldrich (St. Louis, MO, USA). The NAP-5 columns (Sephadex G-25 DNA grade) were purchased from Cytiva (Little Chalfont, UK).

### Preparation of ssDNA-AuNPs using freezing method

The probe ssDNA was immobilized on the AuNP surface *via* thiol–Au bond using the freezing method, which enables fast conjugation through freezing–thawing process.^18^ After treatment with DTT and purification using the NAP-5 column, the probe ssDNA was mixed with AuNPs (concentration ratio of 40 000 (DNA) : 1 (AuNP)), and frozen at a temperature of −80 °C for 50 min. In order to control the amount of immobilized probe DNA, EG was added at various concentrations (1, 3, 5 and 8 mM) in the mixture before freezing. After thawing, the solution was centrifuged at speed of 15 000 rpm for 10 min to remove the unreacted probe ssDNA, and the supernatant was replaced with 1 mL of PN buffer (10 mM phosphate buffer, pH 7 and 0.1 M NaCl) including 0.01% Tween20. The washing process was repeated three times, and the ssDNA-AuNPs were re-dispersed in PN buffer at a concentration of 750 pM as a stock solution.

MCH was modified to ssDNA-AuNPs as follows. The stock ssDNA-AuNP solution (750 pM) was incubated with 1 mM MCH at a temperature of 50 °C for 10 or 60 min. To remove the unreacted MCH, the solution was centrifuged at 15 000 rpm for 10 min, and the supernatant was replaced with 1 mL of PN buffer containing 0.01% Tween20. The washing process was repeated three times, and finally, the ssDNA-AuNPs were re-dispersed in PN buffer at a concentration of 750 pM as a stock solution. The amount of immobilized probe ssDNA was estimated as previously described.^14^ Briefly, 10 mM DTT was added to the ssDNA-AuNP solution and incubated at 25 °C for 48 h. After centrifugation at a speed of 15 000 rpm for 10 min, the concentration of the released probe ssDNA in the supernatant was quantified with the QuantiFluor ssDNA System (Promega, Madison, WI, USA) using non-thiolated ssDNA as a standard. The amount of immobilized ssDNA per particle was calculated using the AuNP concentration obtained from the absorbance at 530 nm.

### Colorimetric detection of the target ssDNA using ssDNA-AuNPs

Various concentrations (0–75 nM) of the target ssDNA (4 μL) were added to 500 pM ssDNA-AuNP solution (14 μL) and incubated for 10 min at room temperature. After the incubation, 5 M NaCl (2 μL) was added to the sample and incubated for 60 min. Images were taken with a digital camera, and digital colour analysis of the solution colour images was performed using the ImageJ software.^27^ In brief, the images were split into red, green and blue components. The redness of the solution colour was quantitatively evaluated by calculating the redness value (red − (green + blue)/2) from the intensity of each colour component as described.^15^ It was also confirmed in advance that the redness values could reflect the change in the colour of AuNP solution upon aggregation as the OD_630_/OD_530_ ratio obtained from UV-VIS spectra (ESI, Fig. S1†).

### Colorimetric evaluation of dispersion stability of ssDNA-AuNPs against salt

Various concentrations (0–5 M) of NaCl (6 μL) were added to 500 pM ssDNA-AuNP solution (4 μL) and incubated for 1 h at room temperature. Images were taken with a digital camera, and the RGB analysis of solution colour images was performed as described above.

### Zeta potential of the ssDNA-AuNPs

AuNPs and ssDNA-AuNPs were diluted with MQ water. The size and zeta potential of 75 pM unmodified AuNPs and ssDNA-AuNPs were evaluated using a Zetasizer-Nano ZS (Malvern Worcestershire, UK).

## Result and discussion

### Preparation of ssDNA-AuNPs with controlled amount of immobilized ssDNA

**Fig. 1:**
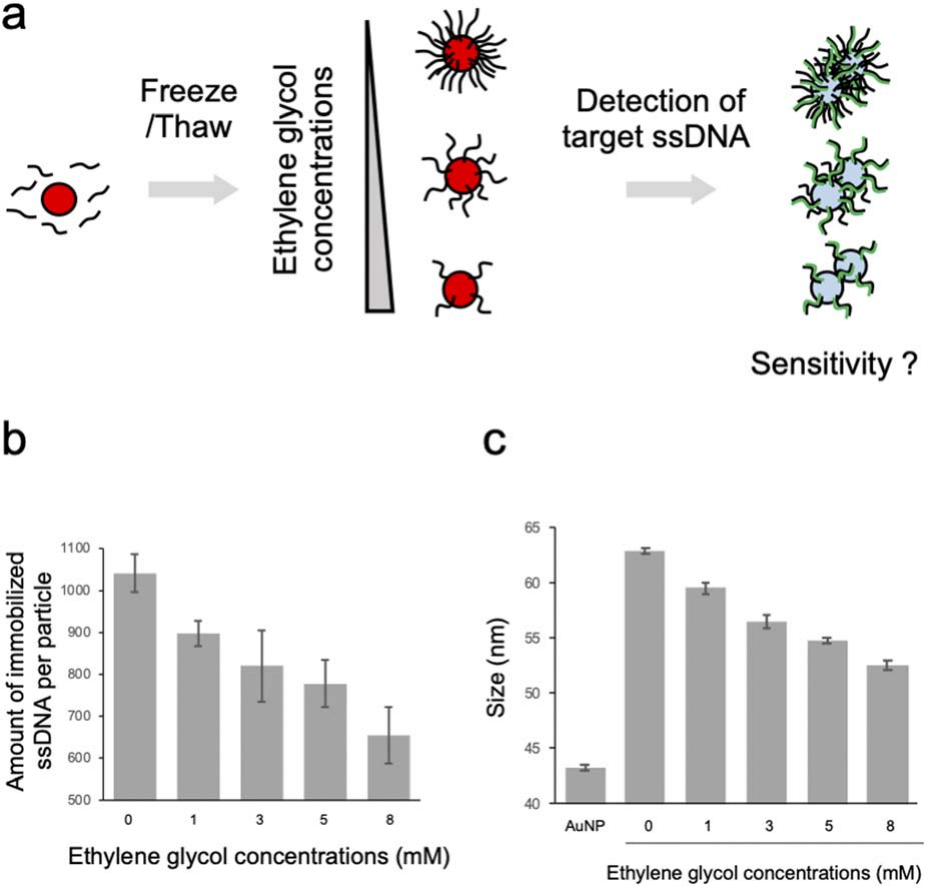
Preparation of ssDNA-AuNPs with controlled amount of immobilized DNA using EG. (a) Schematic illustration of the synthesis of ssDNA-AuNPs with the controlled amount of immobilized ssDNA with the freezing method using EG and the detection of the target ssDNA. (b) Effect of EG on the amount of ssDNA immobilized on AuNPs. The averaged values of the three independent measurements were shown. (c) Effect of EG on the size of ssDNA-AuNPs synthesized in the presence of EG. The averaged values of the three measurements were shown.

The probe ssDNA was immobilized on the surface of AuNPs *via* thiol-Au bonds using the freezing method (Fig. 1a). We first examined the effect of EG on the density of the immobilized probe ssDNA. As shown in Fig. 1b, the amount of immobilized ssDNA was decreased in a dose dependent manner of EG. It is plausible that the inhibition of ice crystal formation by EG during the freezing process may cause differences in the degree of enrichment of AuNPs and the probe ssDNA, and the amount of immobilized ssDNA on the surface of AuNPs could be controlled.

The particle size of ssDNA-AuNPs was then measured by DLS to evaluate the immobilization of the probe ssDNA on the surface of the AuNPs (Fig. 1c). When the probe ssDNA was immobilized without EG, the particle size was estimated to be approximately 63 nm. This result suggests that the ssDNA immobilized at the highest density has an upright structure considering the calculated length of the thiolated 18mer ssDNA (approximately 10 nm). As shown in the figure, the particle size was decreased in a dose dependent manner of EG. Taken together with the result of Fig. 1b, this result indicates that the size was decreased by when lower amount of ssDNA was immobilized. These results are consistent with the previous study on showing that the thickness of immobilized ssDNA on a solid surface was decreased when lower amount of ssDNA was immobilized.^28,29^ It is plausible that ssDNA immobilized on AuNPs at lower densities has a more randomly oriented structure, whereas ssDNA immobilized at higher densities has an almost upright structure, as discussed for the ssDNA immobilized on a solid surface.^28,29^ For comparison, the size of AuNPs modified with non-thiolated ssDNA (ssDNA_adsorbed_-AuNP) was estimated by DLS (ESI, Fig. S2†). The size of ssDNA_adsorbed_-AuNP was approximately 47 nm. This result is consistent with the previous report showing that the size of AuNP was increased by 7 nm due to the adsorption of ssDNA,^30^ supporting that thiolated ssDNA was immobilized by thiol-Au bonds, not by physical adsorption.

### Effect of DNA density on the detection of the target ssDNA using ssDNA-AuNPs

**Fig. 2:**
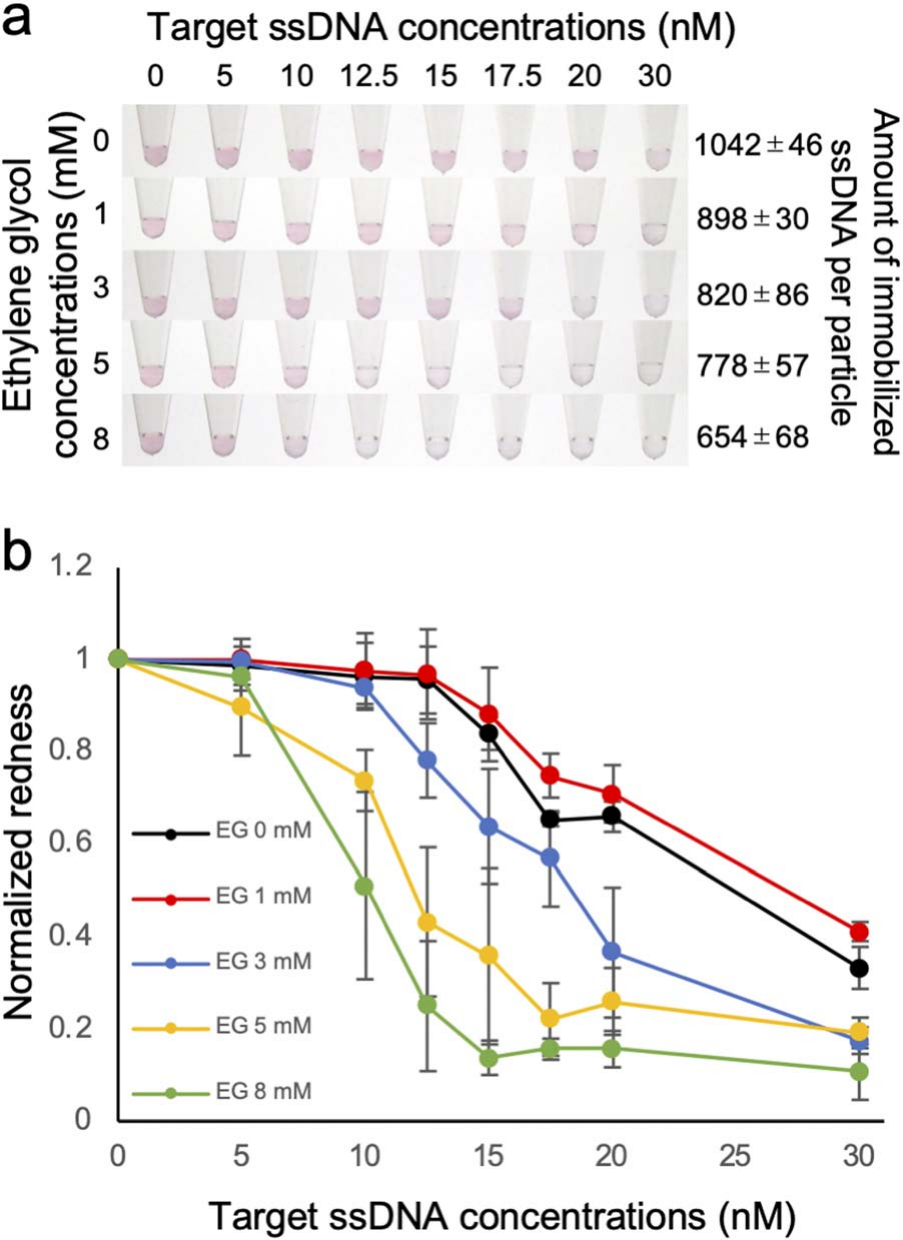
Effect of the amount of immobilized ssDNA on the detection sensitivity of the target ssDNA using ssDNA-AuNPs. (a) Analysis of the colour of AuNP solutions when subjected to increasing concentrations of the target ssDNA. The ssDNA-AuNP samples synthesized in the presence of different concentrations of EG (shown at left) was shown. The number of immobilized ssDNA per particles was also shown (right). (b) Normalized redness values obtained from the solution colour. The redness value of the samples without target ssDNA was normalized as 1.0. The averaged values of three different samples tubes were shown.

Effect of the density of immobilized ssDNA on the detection of the target ssDNA was then evaluated by observing the colour change of the solution, which is due to the aggregation of ssDNA-AuNP induced by the hybridization of the target ssDNA in the presence of salt (Fig. 2). As shown in Fig. 2a, the solution colour was changed from red to blue/transparent when higher amount of target ssDNA was added. Interestingly, significant colour change at lower amount of target ssDNA was observed for the AuNP samples with lower amount of immobilized ssDNA. This result clearly indicated that the detection sensitivity was better for the AuNPs with lower DNA density. Fig. 2b showed the redness values obtained from the sample tube images, supporting the better detection sensitivity for the samples with lower DNA density. Significant difference between the data was confirmed by one-way ANOVA, then the *post hoc* Turkey test was performed. The lowest concentrations that showed significant difference (*p* < 0.05) from the data without the target ssDNA were determined as limit of detection (LOD); 17.5 nM (0 mM EG), 12.5 nM (3 mM EG) and 10 nM (5 and 8 mM EG), respectively (ESI, Fig. S3†), supporting that LOD was improved for the samples with higher EG concentrations (corresponding to lower amount of immobilized ssDNA). The increased sensitivity of target ssDNA detection might be due to the increased ratio of dsDNA on the AuNP surface. Sekine *et al.* reported the attraction force between the blunt-ends of immobilized dsDNA.^31^ Thus it is plausible that the attraction force between particles was increased for the AuNPs with lower amount of immobilized ssDNA.

### Effect of DNA density on the salt stability of ssDNA-AuNPs

**Fig. 3:**
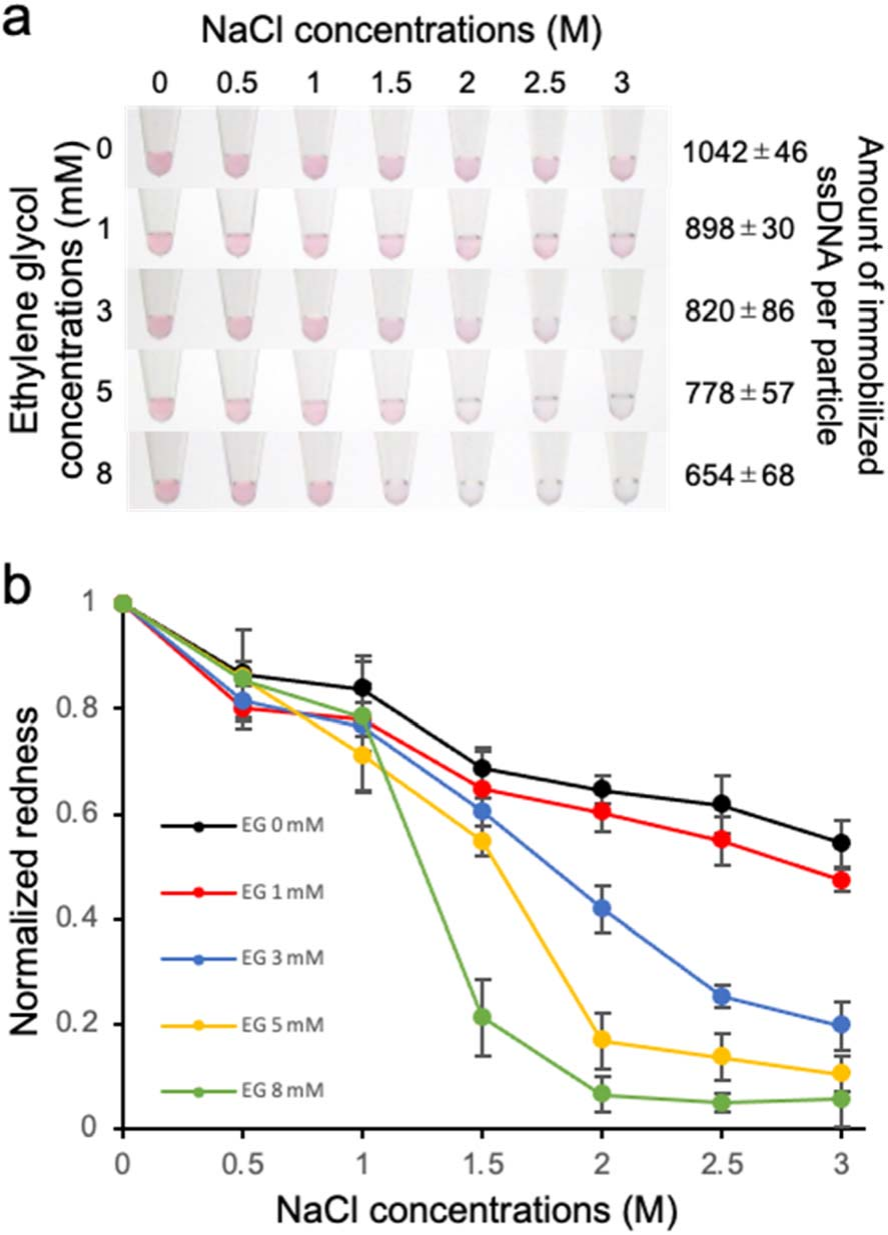
Effect of the amount of immobilized ss DNA on the dispersion stability against salt. (a) Analysis of the colour of AuNP solutions when subjected to increasing concentrations of NaCl. The ssDNA-AuNP samples synthesized in the presence of different concentrations of EG (shown at left) was shown. The number of immobilized ssDNA per particles was also shown (right). (b) Normalized redness values obtained from the solution colour. The redness value of the samples without NaCl was normalized as 1.0. The averaged values of three different samples tubes were shown.

The dispersion stability of ssDNA-AuNPs against salt was also evaluated. Various concentrations of NaCl was added to the ssDNA-AuNP solutions, and the dispersion stability was evaluated by observing the change in the solution colour due to aggregation (Fig. 3a). As shown in the figure, the NaCl concentration at which the change in the solution colour occurred was decreased when lower amount of the probe ssDNA was immobilized. This result indicates that the dispersion stability of ssDNA-AuNPs in response to the addition of NaCl decreased for the AuNPs with lower amount of immobilized ssDNA. The colour change due to AuNP aggregation was also quantitatively evaluated by calculating the redness value (Fig. 3b), supporting the finding above.

To discuss this result, the zeta potential of the ssDNA-AuNPs was measured. However, no significant difference was observed among the ssDNA-AuNP samples of different DNA densities (ESI, Fig. S3†). This result suggests that the surface charge of ssDNA-AuNPs may not have a significant effect in dispersion stability against NaCl, indirectly suggesting that the steric repulsion derived from immobilized ssDNA, which would be different according to the DNA density, may affect the stability of ssDNA-AuNPs. This is consistent with the previous study showing that steric stabilization related to the DNA layer thickness is the major contributor to the stability of ssDNA-AuNPs.^32^ It is also noted that the particle size was decreased when the number of immobilized ssDNA decreased (Fig. 1c), supporting that the size of ssDNA-AuNPs and the stability against salt could be correlated.

### Sensitivity of target ssDNA detection using MCH-modified ssDNA-AuNPs

**Fig. 4:**
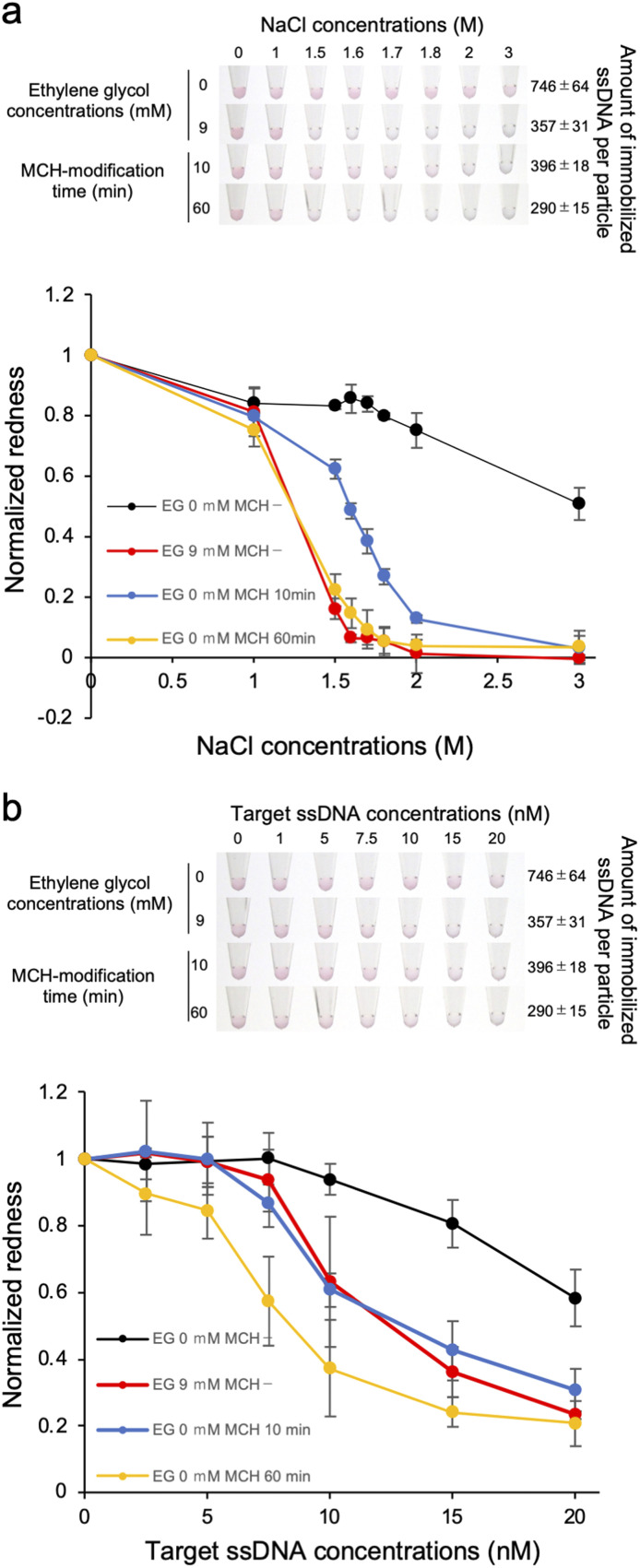
Effect of the MCH modification of ssDNA-AuNP on the dispersion stability against salt (a) and the detection sensitivity of the target ssDNA (b). (a) The solution colour (upper) and the redness value (lower) of MCH-modified ssDNA-AuNPs when subjected to increasing concentrations of NaCl. The ssDNA-AuNP samples synthesized in the absence or presence of 9 mM EG and ones modified with MCH at different time length (10 and 60 min) were used. The number of immobilized ssDNA per particles was also shown (right). The redness value of the samples without NaCl was normalized as 1.0. The averaged values of three different samples tubes were shown. (b) Analysis of the colour of AuNP solutions when subjected to increasing concentrations of the target ssDNA. The redness value of the samples without the target ssDNA was normalized as 1.0.

The results above indicate that the detection sensitivity of the target ssDNA was better when lower amount of the probe ssDNA was immobilized on the AuNP surface. The dispersion stability was also decreased for the samples with lower amount of immobilized ssDNA. Thus it is still unclear if the improvement of the detection sensitivity is due to lower DNA density solely or the decreased stability against salt. Although these are not mutually exclusive, we attempted to discuss the reason by producing ssDNA-AuNPs with different amount of immobilized ssDNA but with similar dispersion stability again salt. For this purpose, MCH was used for surface modification to improve the dispersion stability of ssDNA-AuNPs.^24,25^ First, we investigated the dispersion stability against NaCl of ssDNA-AuNPs produced at different EG concentrations and MCH treatment time length. The number of the immobilized probe ssDNA was reduced by the treatment time with MCH, and EG was also added to reduce the number of the immobilized probe ssDNA. Fig. 4a showed the solution colour of ssDNA-AuNPs produced with/without MCH modification (upper), and the redness value obtained from the pictures (lower). The number of the immobilized ssDNA was quantified as described above. As shown in the figure, we could successfully obtain two types of ssDNA-AuNPs that have similar dispersion stability against NaCl but have different numbers of immobilized ssDNA (-MCH, 9 mM EG, 357 ± 31 ssDNAs per particle; +MCH 60 min, 290 ± 15 ssDNAs per particle). Then the detection sensitivity of the target ssDNA was investigated (Fig. 4b). Importantly, ssDNA-AuNPs with lower DNA density (+MCH 60 min, yellow line) showed aggregation at addition of lower amount of the target ssDNA compared with the sample with higher DNA density of the similar salt dispersion stability against salt (-MCH, 9 mM EG, red line), indicating that the detection sensitivity is better for ssDNA-AuNPs of lower DNA density. This observation might be due to the increase in the ratio of dsDNA on the AuNP surface, leading to the increase in the attraction force between blunt-end on the AuNP surface. Taken all together, better sensitivity for the ssDNA-AuNPs with the lower DNA density shown in Fig. 2 could be due to the number of immobilized ssDNA, not by the decrease in the dispersion stability against salt.

## Conclusions

In this study, we developed a new method to control the density of immobilized ssDNA using EG in the freezing method for the synthesis of ssDNA-AuNPs, and investigated the effect of the DNA density on the detection sensitivity of the target ssDNA. The sensitivity was better for the ssDNA-AuNPs with lower DNA density. While evaluating the dispersion stability against salt, we observed a decrease in dispersion stability with decreasing DNA density. To investigate the reason for the increased detection sensitivity, we modified the ssDNA-AuNPs with MCH for better dispersion stability against salt. The results suggest that the DNA density, rather than the dispersion stability, has a significant impact on detection sensitivity. These results indicate that the sensitivity of the target ssDNA can be improved by controlling the DNA density of ssDNA-AuNPs. Further enhancement of sensitivity using various DNA-modified AuNPs can be expected in the future.

## Author contributions

T. Z. conceived the project. T. Z., G. H., N. F., A. O., T. A. and M. M. designed the experiments. G. H. and N. F. performed the experiments. G. H. and T. Z. wrote the initial manuscript, and all the authors discussed the results and contributed to the manuscript preparation.

## Conflicts of interest

There are no conflicts to declare.

## Supplementary Material

RA-013-D3RA06528F-s001
