# Correction: Effect of DNA density immobilized on gold nanoparticles on nucleic acid detection

**DOI:** 10.1039/d3ra90106h

**Published:** 2023-11-03

**Authors:** Gen Hirao, Nanami Fukuzumi, Atsushi Ogawa, Tsuyoshi Asahi, Mizuo Maeda, Tamotsu Zako

**Affiliations:** a Department of Chemistry and Biology, Graduate School of Science and Engineering, Ehime University 2-5 Bunkyo Matsuyama Ehime 790-8577 Japan zako.tamotsu.us@ehime-u.ac.jp; b Proteo-Science Center, Ehime University 2-5 Bunkyo Matsuyama Ehime 790-8577 Japan; c RIKEN Cluster for Pioneering Research 2-1 Hirosawa, Wako Saitama 351-0198 Japan

## Abstract

Correction for ‘Effect of DNA density immobilized on gold nanoparticles on nucleic acid detection’ by Gen Hirao *et al.*, *RSC Adv.*, 2023, **13**, 30690–30695, https://doi.org/10.1039/D3RA06528F.

The authors regret that the name of one of the authors (Mizuo Maeda) was shown incorrectly in the original article. The corrected author list is as shown above.

The Royal Society of Chemistry apologises for these errors and any consequent inconvenience to authors and readers.

## Supplementary Material

