# Supplementary material for: Effect of DNA density immobilized on gold nanoparticles on nucleic acid detection
Source: RSC Adv. 2023 Oct 19;13(44):30690–5. doi: 10.1039/d3ra06528f (PMC10585452; doi:10.1039/d3ra06528f)
Supplement: RA-013-D3RA06528F-s001 [file RA-013-D3RA06528F-s001.pdf]

Electronic Supplementary Material (ESI) for RSC Advances.
This journal is © The Royal Society of Chemistry 2023

## Supplementary Information

# Effect of DNA density immobilized on gold nanoparticles on nucleic acid detection

Gen Hirao,[a] Nanami Fukuzumi, [a] Atsushi Ogawa, [b] Tsuyoshi Asahi, [a] Mizuo Maeda [c] and Tamotsu Zako [*a]

[a] *Department of Chemistry and Biology, Graduate School of Science and Engineering, Ehime University, 2-5 Bunkyo, Matsuyama, Ehime 790-8577 Japan E-mail:zako.tamotsu.us@ehime-u.ac.jp*
[b] *Proteo-Science Center, Ehime University, 2-5 Bunkyo, Matsuyama, Ehime 790-8577, Japan*
[c] *RIKEN Cluster for Pioneering Research, 2-1 Hirosawa, Wako, Saitama 351-0198, Japan*

## Table of Contents

**Fig. S1** Estimation of the colour change of AuNP solution using the redness value.

**Fig. S2** Size of AuNPs modified with non-thiolated ssDNA ($ssDNA_{adsorbed}$-AuNP)

**Fig. S3** Redness values obtained from the solution color of ssDNA-AuNPs with different amount of immobilized ssDNA after addition of target ssDNA samples.

**Fig. S4** Zeta potential of ssDNA-AuNPs with controlled amount of immobilized DNA using EG

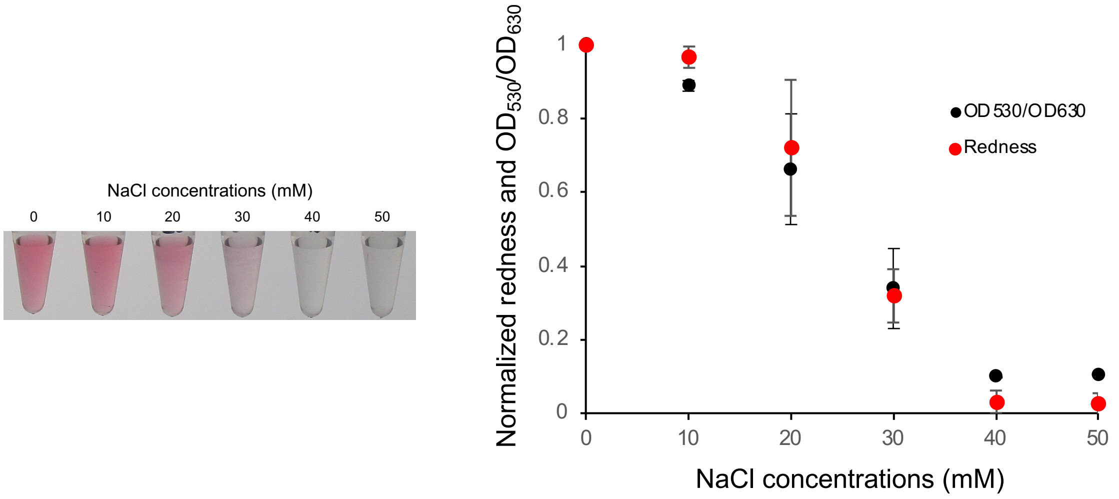


**Fig. S1** Estimation of the color change of AuNP solution using the redness value. Aggregation of AuNP was induced by the addition of NaCl at various concentrations (left). The colour changes of the AuNP solutions were estimated by the redness values obtained from the images and the $OD_{530}/OD_{630}$ ratio obtained from UV-VIS spectra (right). The redness value and the $OD_{530}/OD_{630}$ ratio value of the samples without NaCl were normalized as 1.0.

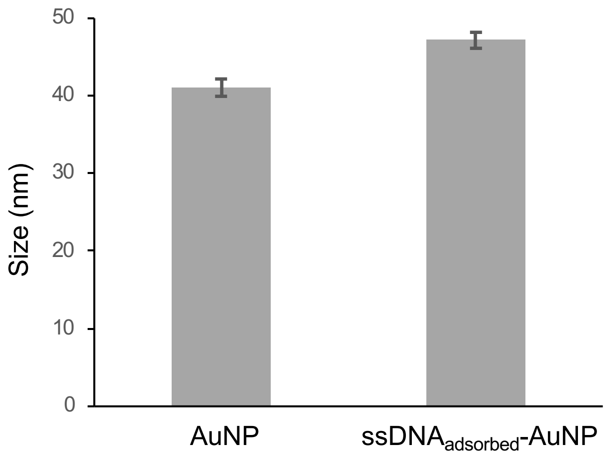


**Fig. S2** Size of AuNPs modified with non-thiolated ssDNA (ssDNA$_{adsorbed}$-AuNP). AuNPs were mixed with non thiolated ssDNA (ratio of 40000 (DNA) :1 (AuNP)), and the mixture was incubated at room temperature for 30 min. The size of 75 pM unmodified AuNPs and ssDNA$_{adsorbed}$-AuNP diluted in MQ water was evaluated using a Zetasizer-Nano ZS (Malvern Worcestershire, UK). The averaged values of the three measurements were 41.1±0.3 for AuNP and 47.2±0.6 for ssDNA$_{adsorbed}$-AuNP, respectively.

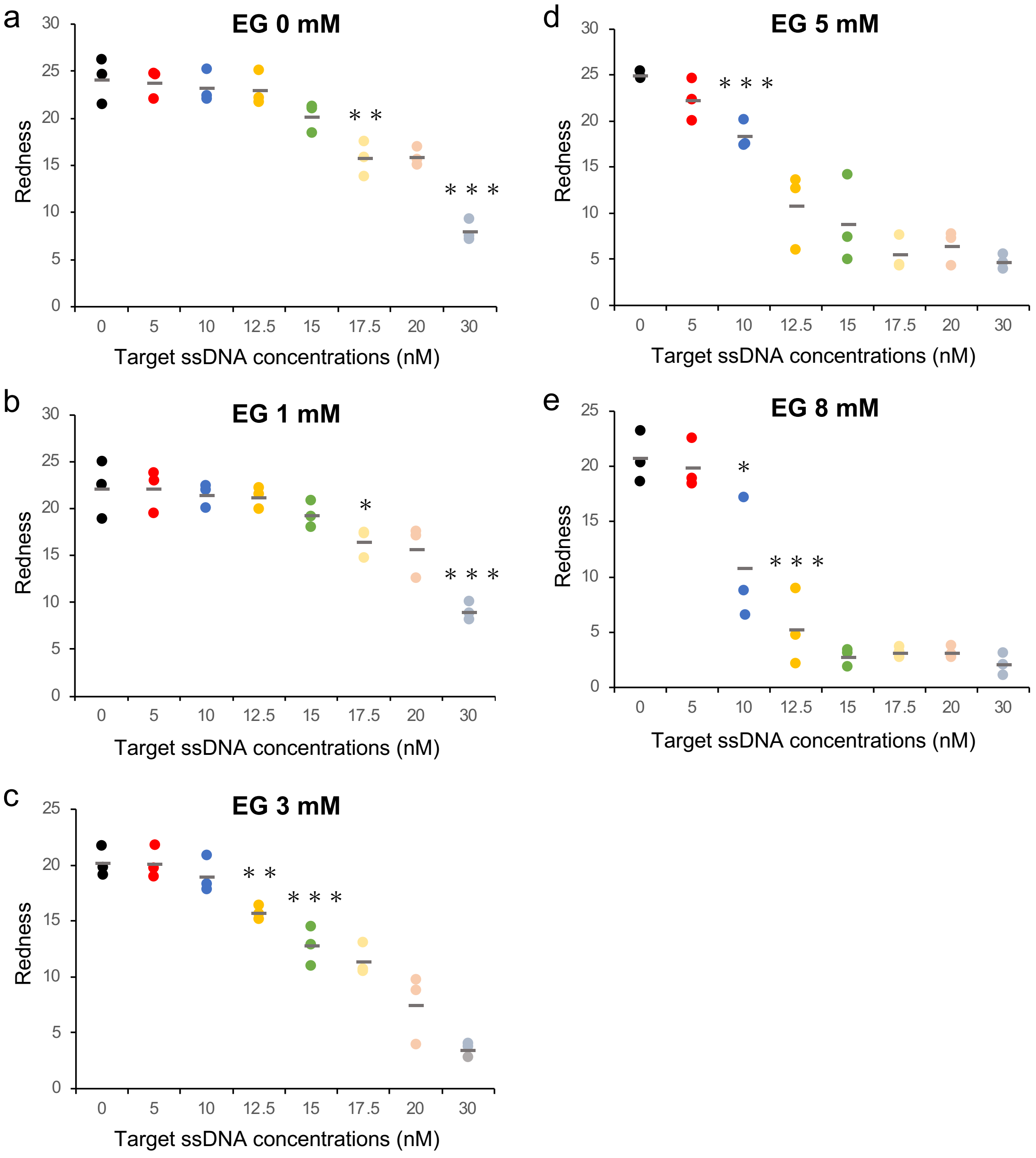


**Fig. S3** Redness values obtained from the solution colour of ssDNA-AuNPs with different amount of immobilized ssDNA after addition of target ssDNA samples (shown in Fig. 2a). The values of three different samples tubes and average values (black line) were shown. The lowest target ssDNA concentrations that showed significant difference ($^{*}p<0.05$, $^{**}p<0.01$ or $^{***}p<0.001$) from the data without the target DNA were marked.

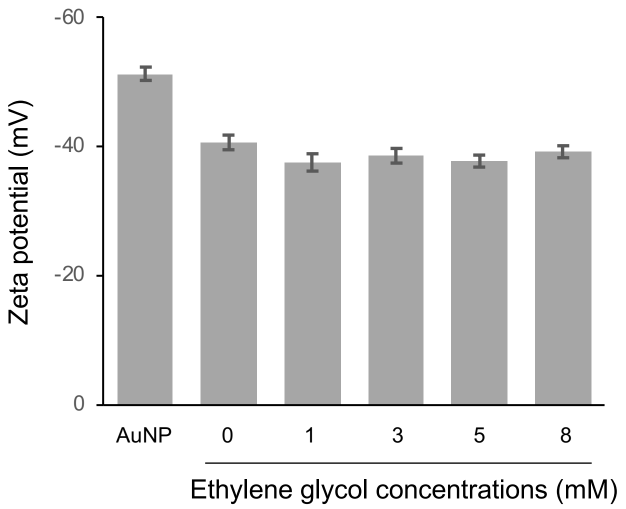


**Fig. S4** Zeta potential of ssDNA-AuNPs with controlled amount of immobilized DNA using EG. Zeta potential values of 75 pM unmodified AuNPs and ssDNA-AuNPs with controlled amount of immobilized DNA using EG were evaluated using a Zetasizer-Nano ZS (Malvern Worcestershire, UK). The averaged values of the three measurements were shown.
